# Atrial fibrillation burden and symptom, quality of life, and healthcare resource utilization after cryoballoon ablation in persistent atrial fibrillation

**DOI:** 10.1093/europace/euaf150

**Published:** 2025-08-29

**Authors:** Muhan Yeo, So-Ryoung Lee, JungMin Choi, Kyung-Yeon Lee, Hyo-Jeong Ahn, Soonil Kwon, Ji Hyun Lee, Youngjin Cho, Il-Young Oh, Hong-Euy Lim, Min-Soo Cho, Gi-Byoung Nam, Seil Oh, Young-Keun On, Eue-Keun Choi

**Affiliations:** Department of Internal Medicine, Seoul National University College of Medicine, 101 Daehak-ro, Jongno-gu, Seoul 03080, Republic of Korea; Department of Internal Medicine, Seoul National University College of Medicine, 101 Daehak-ro, Jongno-gu, Seoul 03080, Republic of Korea; Department of Internal Medicine, Seoul National University Hospital, Seoul, Republic of Korea; Department of Internal Medicine, Seoul National University Hospital, Seoul, Republic of Korea; Department of Internal Medicine, Seoul National University Hospital, Seoul, Republic of Korea; Department of Internal Medicine, Seoul National University Hospital, Seoul, Republic of Korea; Department of Internal Medicine, SMG-SNU Boramae Medical Center, Seoul, Korea; Department of Internal Medicine, Seoul National University Bundang Hospital, Seongnam, Republic of Korea; Department of Internal Medicine, Seoul National University Bundang Hospital, Seongnam, Republic of Korea; Department of Internal Medicine, Seoul National University Bundang Hospital, Seongnam, Republic of Korea; Division of Cardiology, Chung-Ang University Gwangmyeong Hospital, Chung-Ang University College of Medicine, Gwangmyeong-si, Republic of Korea; Division of Cardiology, Asan Medical Center, University of Ulsan College of Medicine, Seoul, Republic of Korea; Division of Cardiology, Asan Medical Center, University of Ulsan College of Medicine, Seoul, Republic of Korea; Department of Internal Medicine, Seoul National University College of Medicine, 101 Daehak-ro, Jongno-gu, Seoul 03080, Republic of Korea; Department of Internal Medicine, Seoul National University Hospital, Seoul, Republic of Korea; Division of Cardiology, Department of Medicine, Cardiac Arrhythmia Center, Heart Vascular and Stroke Institute, Samsung Medical Center, Sungkyunkwan University School of Medicine, 81 Irwon-ro, Gangnam-gu, Seoul 06351, Republic of Korea; Department of Internal Medicine, Seoul National University College of Medicine, 101 Daehak-ro, Jongno-gu, Seoul 03080, Republic of Korea; Department of Internal Medicine, Seoul National University Hospital, Seoul, Republic of Korea

**Keywords:** Atrial fibrillation, Cryoablation, Implantable Electrode, Quality of Life

## Abstract

**Aims:**

To investigate the relationship between continuously monitored atrial fibrillation (AF) burden after cryoballoon ablation (CBA) and improvements in AF-related symptoms, quality of life (QoL), and AF-related healthcare resource utilization (HCRU) in early persistent AF.

**Methods and results:**

This secondary analysis of the multicentre COOL-PER trial included patients with early persistent AF who underwent CBA and continuous monitoring via an implantable loop recorder. Post-CBA AF burden was defined as the percentage of time in AF between 9- and 12-month after CBA, categorized as <0.1%, 0.1 to <10%, and ≥10%. Symptom improvement was assessed using the European Heart Rhythm Association symptom score and QoL with the SF-36 survey. AF-related HCRU was defined as rhythm control interventions after the 90-day blanking period and cardiovascular-related hospitalizations or emergency room visits. Among 130 patients (mean baseline AF burden 77 ± 34%), AF burden significantly decreased post-CBA, with 50.0% achieving <0.1%, 28.5% in 0.1 to <10%, and 21.5% ≥ 10%. Symptom improvement rate was highest in the <0.1% group (89.2%), followed by the 0.1 to <10% (78.4%) and ≥10% (46.4%) groups (*P* < 0.001). Significant QoL improvement was observed in the <0.1% and 0.1 to <10% groups but not in the ≥10% group. Rhythm control interventions and cardiovascular-related hospitalizations or emergency room visits were more frequent in the ≥10% group (29%, 43%) than in the 0.1 to <10% (5.4%, 22%) and <0.1% (1.5%, 7.7%) groups (both *P* < 0.001).

**Conclusion:**

In early persistent AF, lower AF burden 1-year post-CBA was associated with greater AF-related symptom improvement, significant QoL enhancement, and reduced HCRU during follow-up.

## Graphical Abstract

**Graphical Abstract:**
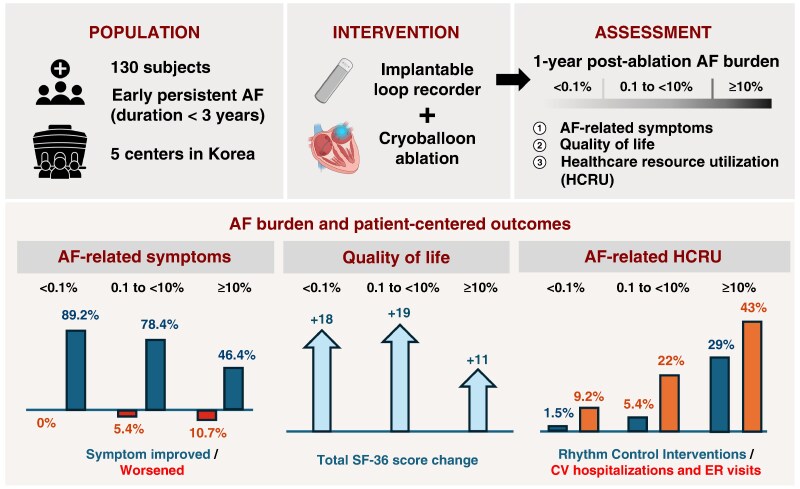

What’s New?

- This study demonstrates a quantitative relationship between implantable loop recorder (ILR)-monitored post-cryoballoon ablation atrial fibrillation (AF) burden and patient-centred outcomes in early persistent AF patients.
- Achieving AF burden below 10% (particularly below 0.1%) at one year was significantly associated with greater symptom improvement and quality-of-life enhancement.
- Patients with lower post-ablation AF burden had substantially fewer rhythm control interventions and cardiovascular-related hospitalization and emergency room visits during follow-up.
- While complete AF elimination remains ideal, our findings suggest that substantial AF burden reduction should be considered a clinically meaningful treatment success.
- AF burden represents a quantitative and actionable treatment target beyond traditional binary assessment of ablation success in early persistent AF management.

## Introduction

Catheter ablation has become an established long-term rhythm control strategy for managing atrial fibrillation (AF),^1,2^ demonstrating significant benefits in reducing AF-related symptoms and improving quality of life (QoL) in both paroxysmal and persistent AF.^3,4^ The mainstay of catheter ablation in AF is pulmonary vein isolation (PVI),^2,5^ with cryoballoon ablation (CBA) demonstrating notable benefits, particularly in reducing AF recurrence for patients who are either intolerant to antiarrhythmic drugs (AAD) or have paroxysmal AF as a first-line approach.^6–8^

Prior studies using continuous cardiac monitoring reported that CBA in patients with paroxysmal AF reduced progression to persistent AF and AF burden.^9,10^ AF burden itself has recently been highlighted as a strong predictor of cardiovascular outcomes and mortality.^11,12^ Specifically, post-ablation atrial arrhythmia burden offers a more accurate prediction of clinical outcomes and QoL improvements than simply recording atrial arrhythmia recurrence.^12–15^ However, most previous studies have assessed AF burden using noninvasive, intermittent rhythm monitoring, which is less accurate than continuous monitoring and may misrepresent the true AF burden.^15,16^

Recent research using implantable loop recorder (ILR) monitoring showed that in patients with early persistent AF, CBA significantly reduced AF burden with symptom and QoL improvement.^17^ Furthermore, it revealed that approximately 25% of patients clinically classified as having persistent AF were actually having high-burden paroxysmal AF based on pre-ablation ILR results, with a median pre-ablation burden of 24%.^17^ This finding highlights the substantial heterogeneity within clinically diagnosed persistent AF. However, data remain limited regarding how continuously monitored post-ablation AF burden correlates with symptoms, QoL, and arrhythmia-specific healthcare resource utilization (HCRU) along with this heterogeneity of persistent AF.

Accordingly, we aimed to investigate the relationship between the continuously monitored post-ablation AF burden after CBA and outcomes, including AF-related symptoms, QoL, and AF-related HCRU, in patients with early persistent AF.

## Methods

This study is a secondary analysis based on data from the COOL-PER trial, a multicentre, investigator-initiated, single-arm, and prospective study conducted across five sites in Korea. The COOL-PER trial evaluated patients with early persistent AF, defined as clinically persistent AF lasting no more than three years, who underwent PVI using CBA.^17^ Each participant received an ILR (Reveal LINQ; Medtronic, Inc.), implanted within 7–90 days before ablation to enable continuous cardiac rhythm monitoring. AF burden was defined as the percentage of time spent in AF.^18,19^ Procedural details of CBA and ILR settings have been previously published^17^ and can also be found in Supplementary material online, *Table S1*. While AAD cessation was recommended after a 90-day post-ablation blanking period, they could be continued or adjusted at the treating physician's discretion during the follow-up. Similarly, oral anticoagulants (OACs) were prescribed for all patients for 2 months post-CBA and were either continued or discontinued at the physician’s discretion. Informed consent was obtained from all participants, and the study adhered to ethical guidelines established in the Declaration of Helsinki. The COOL-PER trial was approved by the relevant ethics and institutional review boards and registered on ClinicalTrials.gov (NCT05507749).

### Patient selection

Eligibility criteria included patients aged 20–80 years with symptomatic, drug-refractory persistent AF documented within the preceding three years. Persistent AF was defined as continuous AF episodes persisting for at least seven days, confirmed by electrocardiogram (ECG) and Holter monitoring, with a history of requiring cardioversion for AF episodes lasting seven days or more.^1^ Major exclusion criteria included long-standing persistent AF lasting more than 3 years, left atrial (LA) diameter exceeding 50 mm, left ventricular ejection fraction (LVEF) below 30%, previous AF ablation or cardiac surgery, recent myocardial infarction or percutaneous coronary intervention within three months, and recent cerebrovascular events within six months. Detailed inclusion and exclusion criteria are available in Supplementary material online, *Table S2*.

### Atrial fibrillation burden analysis and subtypes of persistent atrial fibrillation

ILRs continuously recorded AF episodes, and AF burden at 1-year post-CBA was assessed using the percentage of time in AF episodes between 9- and 12-month periods. Patients were categorized into three groups based on post-CBA 1-year AF burden: < 0.1%, 0.1 to <10%, and ≥10%. The 0.1% and 10% thresholds are equivalent to approximately 1.4 and 144 min of total AF per day, and were selected based on previous studies in which their correlations with clinical outcomes have been shown.^12,13,20^ Pre-CBA subtypes of persistent AF were classified as (i) high-burden paroxysmal AF or (ii) ILR-confirmed persistent AF, based on whether the AF lasted more than 7 days according to the pre-CBA ILR monitoring results.

### Outcomes and follow-up

Follow-up visits were scheduled at intervals of 1, 3, 6, 9, and 12 months (1-year) after ablation. At each follow-up visit, a 12-lead ECG and ILR analysis were performed. We evaluated AF-related symptoms and QoL at baseline and after a 1-year follow-up. AF-related symptoms were assessed using the European Heart Rhythm Association (EHRA) symptom scale, and the symptom changes were categorized as improved, unchanged, and worsened based on changes in the EHRA score. QoL improvements were evaluated using the physical component summary (PCS), mental component summary (MCS), and a total score calculated as their sum, based on the SF-36 survey.^21^ PCS and MCS scores each range from 0 to 100, with higher scores indicating better health status. All scores were measured at baseline and at the 12-month follow-up, and changes between the two time points were assessed.

AF-related HCRU was defined as additional rhythm control interventions and cardiovascular (CV)-related hospitalizations or emergency room (ER) visits. Rhythm control interventions included direct current cardioversion (DCC), redo AF catheter ablation, and AF surgery occurring between the 90-day post-ablation blanking period and the 1-year follow-up. CV-related hospitalizations and ER visits were defined as the presence of any such encounter from the index CBA to 1-year follow-up. AAD and OAC prescription changes across 1-year follow-ups were also assessed. Outcomes were compared across post-CBA AF burden levels.

### Statistical analysis

Categorical variables were expressed as frequencies and percentages, while continuous variables were reported as mean ± standard deviation (SD) or median [1st and 3rd quartiles]. Group comparisons of baseline characteristics and AF-related symptom changes were conducted using chi-square test or Fisher’s exact test, and continuous variables using independent t-tests, one-way analysis of variance (ANOVA), or Kruskal–Wallis test. Differences in AF burden and QoL before and after CBA were assessed using paired *t*-tests or Wilcoxon signed-rank tests, as appropriate based on data distribution. We performed a subgroup analysis of AF-related symptoms, QoL, and AF-related HCRU based on recurrent AF burden in patients with high-burden paroxysmal AF and ILR-confirmed persistent AF, categorized by pre-CBA AF type. Statistical significance was set at a two-tailed *P*-value of <0.05; 95% confidence intervals (CIs) are reported along with *P*-values. All analyses were performed using SPSS® v25, Stata® v18, and R Statistical Software version 4.2.11 for Windows (R Core Team).

## Results

Between January 2021 and July 2022, we screened 150 patients from the initial cohort, with 130 ultimately enrolled in the study after exclusions (see Supplementary material online, *Figure S1*). The mean age was 60.0 ± 9.2 years, 23.8% were female, and the mean CHA_2_DS_2_-VASc score was 1.7 ± 1.3. At baseline, all patients presented with clinically persistent AF; 67.7% had persistent AF from the first diagnosis, while 32.3% experienced progression from paroxysmal to persistent AF. Additionally, 33.8% had previously undergone DCC, though sinus rhythm was not restored in 13.6% of these cases. The ILR data captured a mean AF burden of 77 ± 34% prior to CBA (see Supplementary material online, *Figure S2*). Although all patients were clinically categorized with persistent AF, continuous monitoring revealed that 25.4% had high-burden paroxysmal AF, where AF episodes did not persist beyond seven days, while the remaining 74.6% were confirmed as ILR-confirmed persistent AF, with episodes extending beyond 7 days. The mean AF burden decreased substantially following CBA, from 77 ± 34% to 9 ± 21% at 1-year follow-up (*P* < 0.001). When categorizing 1-year post-CBA AF burden, 65 patients (50%) were classified as having <0.1%, 37 patients (28.5%) fell within the 0.1 to <10% range, and 38 patients (21.5%) had an AF burden of ≥10%. All patients in the <0.1% group had zero AF burden at 1 year in this study.

### Baseline characteristics according to 1-year post-cryoballoon ablation atrial fibrillation burden

*Table 1* presents baseline characteristics of the study population according to 1-year post-CBA AF burden. There was no statistically significant difference in pre-CBA AF burden among the <0.1% (71.8 ± 38.2%), 0.1 to <10% (77.0%±31.7%), and ≥10% (90.9%±21.4%) groups (*P* = 0.5). Mean 1-year post-CBA AF burden was 0.0%, 1.3%±2.1%, and 58.6%±34.5% in the <0.1%, 0.1 to <10%, and ≥10% groups, respectively. All three groups showed a significant reduction in AF burden post-CBA compared to pre-CBA (all *P* < 0.001), but the ≥10% group exhibited the smallest reduction (−71.8 ± 38.2%, −75.6%±31.5%, and −32.3%±37.7% in the <0.1%, 0.1 to <10%, and ≥10% groups, respectively, *P* < 0.001). Age, BMI, and CHA_2_DS_2_-VASc score distribution were similar among the three groups. For the pre-CBA AF subtype, the ILR-confirmed persistent AF subtype exhibited a higher proportion in the ≥10% group (93%) than in the 0.1 to <10% (76%) and <0.1% (66%) groups (*P* = 0.025). In echocardiography, LA diameter and volume, and LVEF were not significantly different among the three groups. At 1-year follow-up, the AAD prescription rate was highest in the ≥10% AF burden group, followed by the 0.1 to <10% group and the <0.1% group in sequential order; however, the differences were not statistically significant (71% vs. 65% vs. 50%, *P* = 0.11). The OAC prescription rate at 1 year was higher in the ≥10% group (96%) than in the 0.1 to <10% (72%) and <0.1% (60%) groups (*P* = 0.002).

**Table 1:** Baseline characteristics and outcomes by 1-year post-CBA AF burden.

Variables		Post-CBA AF burden at 12-month after CBA			
	Overall, *N* = 130	<0.1%, *N* = 65	0.1 to <10%, *N* = 37	≥10%, *N* = 28	*P*-value
Baseline characteristics					
Age (years)	60.0 (9.2)	58.9 (10.4)	61.4 (8.2)	60.5 (7.6)	0.5
Sex (female)	31 (24%)	12 (18%)	10 (27%)	9 (32%)	0.3
Body-mass index (kg/m^2^)	25.9 (2.7)	26.2 (2.4)	25.9 (3.3)	25.3 (2.7)	0.3
Actual Pre-CBA AF subtype monitored by ILR					0.025
High-burden paroxysmal AF (type I)	33 (25%)	22 (34%)	9 (24%)	2 (7.1%)	
ILR-confirmed persistent AF (type II)	97 (75%)	43 (66%)	28 (76%)	26 (93%)	
CHA_2_DS_2_-VASc score	1.7 (1.3)	1.7 (1.4)	1.9 (1.2)	1.6 (1.2)	0.5
Days from persistent AF diagnosis	282.4 (209.5)	285.8 (213.3)	219.1 (149.9)	358.0 (245.9)	0.028
History of DC cardioversion before CBA	44 (34%)	24 (37%)	12 (32%)	8 (29%)	0.7
Success	38 (86%)	20 (83%)	10 (83%)	8 (100%)	0.7
Days from DC cardioversion to AF recurrence	83.1 (121.5)	66.7 (107.6)	61.1 (123.3)	151.5 (142.54)	0.2
Echocardiography					
Left atrial diameter (mm)	43.8 (3.9)	43.3 (3.8)	44.5 (4.0)	44.3 (3.6)	0.2
Left atrial volume (ml)	81.7 (21.2)	80.2 (20.8)	81.9 (20.7)	85.2 (23.0)	0.6
Left atrial volume index (ml/m^2^)	44.7 (11.7)	42.8 (11.7)	45.1 (10.9)	48.6 (12.1)	0.063
Left ventricular ejection fraction (%)	57.7 (6.5)	56.7 (7.0)	58.9 (5.8)	58.5 (6.0)	0.3
Pre-CBA ILR burden (%)	77.4 (34.0) 99.8 [51.2, 100.0]	71.8 (38.2) 99.8 [29.1, 100.0]	77.0 (31.7) 99.3 [49.2, 100.0]	90.9 (21.4) 99.9 [95.8, 100.0]	0.5
Baseline EHRA symptom score					>0.9
I	4 (3.1%)	3 (4.6%)	1 (2.7%)	0 (0%)	
IIa	58 (45%)	28 (43%)	16 (43%)	14 (50%)	
IIb	47 (36%)	22 (34%)	14 (38%)	11 (39%)	
III	21 (16%)	12 (18%)	6 (16%)	3 (11%)	
Baseline SF-36 score (PCS + MCS)	139.0 (34.2)	138.3 (32.1)	134.2 (40.3)	146.8 (29.4)	0.4
PCS	70.5 (18.4)	70.8 (17.1)	66.9 (22.2)	74.4 (15.7)	0.4
MCS	68.5 (17.7)	67.4 (16.8)	67.3 (19.6)	72.4 (16.8)	0.4
Medications during follow-up					
AAD use at 12 months after CBA	76 (59%)	32 (50%)	24 (65%)	20 (71%)	0.11
OAC use at 12 months after CBA	90 (71%)	38 (60%)	26 (72%)	26 (96%)	0.002
Healthcare resource utilization					
Rhythm control intervention	11 (8.5%)	1 (1.5%)	2 (5.4%)	8 (29%)	<0.001
DC cardioversion	4 (3.1%)	0 (0%)	0 (0%)	4 (14%)	0.002
Redo AF ablation	6 (4.6%)	1 (1.5%)	1 (2.7%)	4 (14%)	0.035
AF surgery	1 (0.8%)	0 (0%)	1 (2.7%)	0 (0%)	0.5
CV-related hospitalization and ER visit	26 (20%)	6 (9.2%)	8 (22%)	12 (43%)	<0.001
Hospitalization	25 (19%)	5 (7.7%)	8 (22%)	12 (43%)	<0.001
ER visit	2 (1.5%)	1 (1.5%)	0 (0%)	1 (3.6%)	0.5
AF burden, QoL, and AF-related symptom at 12-month follow-up					
Post-CBA AF burden at 12-month after CBA^a^ (%)	13.0 (28.7) 0.1 [0.0, 3.2]	0.0 (0.0) 0.0 [0.0, 0.0]	1.3 (2.1) 0.4 [0.1, 1.6]	58.6 (34.5) 64.8 [20.6, 97.1]	<0.001
EHRA symptom score at 12-month					<0.001
I	90 (69%)	61 (94%)	22 (59%)	7 (25%)	
IIa	23 (18%)	0 (0%)	13 (35%)	10 (36%)	
IIb	15 (12%)	3 (4.6%)	2 (5.4%)	10 (36%)	
III	2 (1.5%)	1 (1.5%)	0 (0%)	1 (3.6%)	
SF-36 score at 12-month (PCS + MCS)	155.5 (25.6)	155.9 (23.4)	153.0 (31.3)	158.1 (22.6)	>0.9
PCS	79.5 (14.9)	80.2 (13.4)	78.1 (18.3)	79.7 (13.7)	>0.9
MCS	76.0 (13.3)	75.7 (12.5)	74.9 (15.4)	78.3 (12.1)	0.7
Change in EHRA symptom score					<0.001
Worsened	5 (3.8%)	0 (0%)	2 (5.4%)	3 (11%)	0.023
Unchanged	25 (19%)	7 (11%)	6 (16%)	12 (43%)	0.001
Improved	100 (77%)	58 (89%)	29 (78%)	13 (46%)	<0.001
Change in SF-36 score (PCS + MCS)	16.9 (31.0)	18.2 (27.2)	18.8 (35.2)	11.2 (33.5)	0.5
PCS	9.2 (17.2)	9.8 (15.9)	11.1 (18.9)	5.3 (17.5)	0.4
PCS (% increased ≥5 points)	74 (58%)	38 (60%)	23 (62%)	13 (46%)	0.4
MCS	7.6 (16.9)	8.4 (14.5)	7.6 (19.9)	5.9 (18.4)	0.7
MCS (% increased ≥5 points)	70 (55%)	35 (56%)	21 (57%)	14 (50%)	0.8
Pre-/post-CBA AF burden change (%)	−64.4 (39.8) −85.9 [−99.9, −26.6]	−71.8 (38.2) −99.8 [−100.0, −29.1]	−75.6 (31.5) −93.2 [−99.5, −47.3]	−32.3 (37.7) −24.1 [−75.8, −0.3]	<0.001

Discrete variables are presented as *n* (%), and continuous variables as mean (SD). AF burden is presented as mean (SD) and median (interquartile range).

AF, atrial fibrillation; CBA, cryoballoon ablation; CV, cardiovascular; DC cardioversion, direct current cardioversion; EHRA, European Heart Rhythm Association; ER, emergency room; ILR, implanatable loop recorder; MCS, mental component score; PCS, physical component score; QoL, quality of life.

^a^AF burden between 9-month and 12-month after CBA.

### Changes in atrial fibrillation–related symptoms and quality of life between baseline and 1-year follow-up according to 1-year post-cryoballoon ablation atrial fibrillation burden

**Figure 1:**
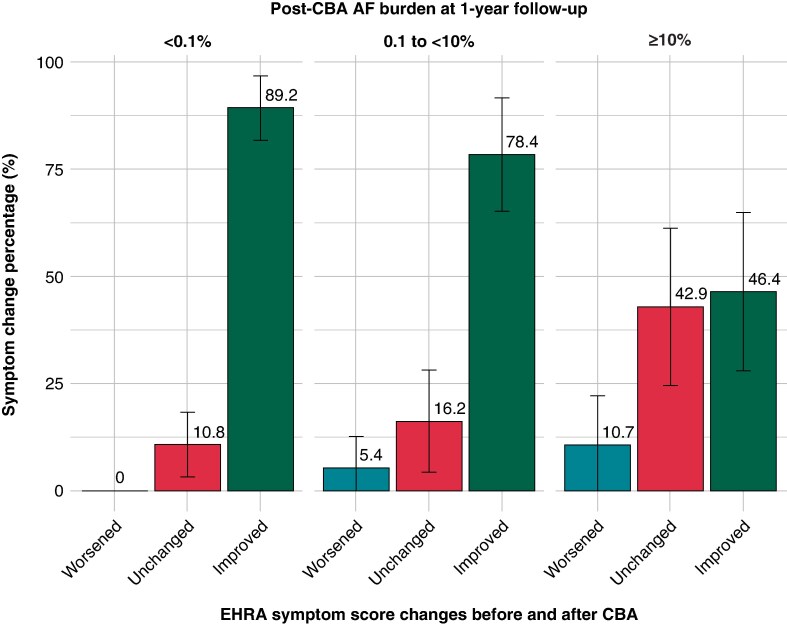
Symptom changes by 1-year post-CBA AF burden. Proportion of patients with improved, unchanged, or worsened atrial fibrillation (AF)-related symptoms from baseline to 1-year follow-up, stratified by post-cryoablation (CBA) AF burden: < 0.1% (*n* = 65), 0.1% to <10% (*n* = 37), and ≥10% (*n* = 28). Symptom improvement, assessed using the EHRA score, was most common in the <0.1% burden group and least in the ≥10% group (*P* < 0.001). The proportion of patients with worsened or unchanged symptoms increased with higher AF burden (*P* = 0.023 and *P* = 0.001, respectively).

Atrial fibrillation–related symptoms, assessed using the EHRA symptom score, improved in 77%, remained unchanged in 19%, and worsened in 3.8% of the total study population. Symptom improvement was highest in the <0.1% post-CBA burden group (89.2%), followed by the 0.1 to <10% group (78.4%) and the ≥10% group (46.4%) (*P* < 0.001, *Figure 1*). Conversely, the proportion of patients with worsened symptoms increased with higher post-CBA burden, observed in 0%, 5.4%, and 10.7% of the <0.1%, 0.1 to <10%, and ≥10% groups, respectively (*P* = 0.023). Similarly, the proportion of patients with unchanged symptoms rose across these groups (10.8%, 16.2%, and 42.9%, *P* = 0.001). The percentage of patients who were symptom-free post-CBA was 94 in the <0.1% group, 59% in the 0.1 to <10% group, and 25 in the ≥10% group.

**Figure 2:**
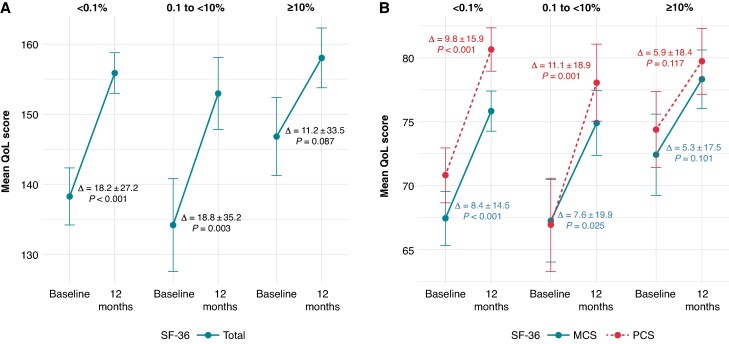
Quality-of-life changes before and after CBA by 1-year post-CBA AF burden. Improvement in quality of life (QoL) as measured by the SF-36 questionnaire, including (*A*) total score, and (*B*) Physical Component Summary (PCS) and Mental Component Summary (MCS) scores. Significant improvements were observed in the <0.1% and 0.1% to <10% AF burden groups (*P* < 0.05 for all measures), while the ≥10% burden group showed no statistically significant changes. Bars represent mean score differences with standard deviations; *P*-values based on paired comparisons from baseline to follow-up within each group.

In the overall study population, pre- to post-CBA QoL, as assessed by the SF-36 survey, showed significant improvement (*Figure 2*). The total SF-36 score increased from 139.0 ± 34.2 to 155.5 ± 25.6 (*P* < 0.001), PCS increased from 70.5 ± 18.4 to 79.5 ± 14.9 (*P* < 0.001), and MCS increased from 68.5 ± 17.7 to 76.0 ± 13.3 (*P* < 0.001). When stratified by post-CBA 1-year AF burden, both the <0.1% and 0.1% to <10% groups showed significant improvements in total SF-36, PCS, and MCS scores. Specifically, total SF-36 increased by 18.2 ± 27.2 (*P* < 0.001) in the <0.1% group and 18.8 ± 35.2 (*P* = 0.003) in the 0.1% to <10% group. PCS increased by 9.8 ± 15.9 (*P* < 0.001) and 11.1 ± 18.9 (*P* < 0.001), respectively, and MCS increased by 8.4 ± 14.5 (*P* = 0.001) and 7.6 ± 19.9 (*P* = 0.025), respectively. However, the ≥10% group did not demonstrate a statistically significant improvement in QoL: total SF-36 changed by 11.2 ± 33.5 (*P* = 0.087), PCS by 5.3 ± 17.5 (*P* = 0.117), and MCS by 5.9 ± 18.4 (*P* = 0.101), respectively.

### Atrial fibrillation–related healthcare resource utilization during follow-up according to post-cryoballoon ablation atrial fibrillation burden

**Figure 3:**
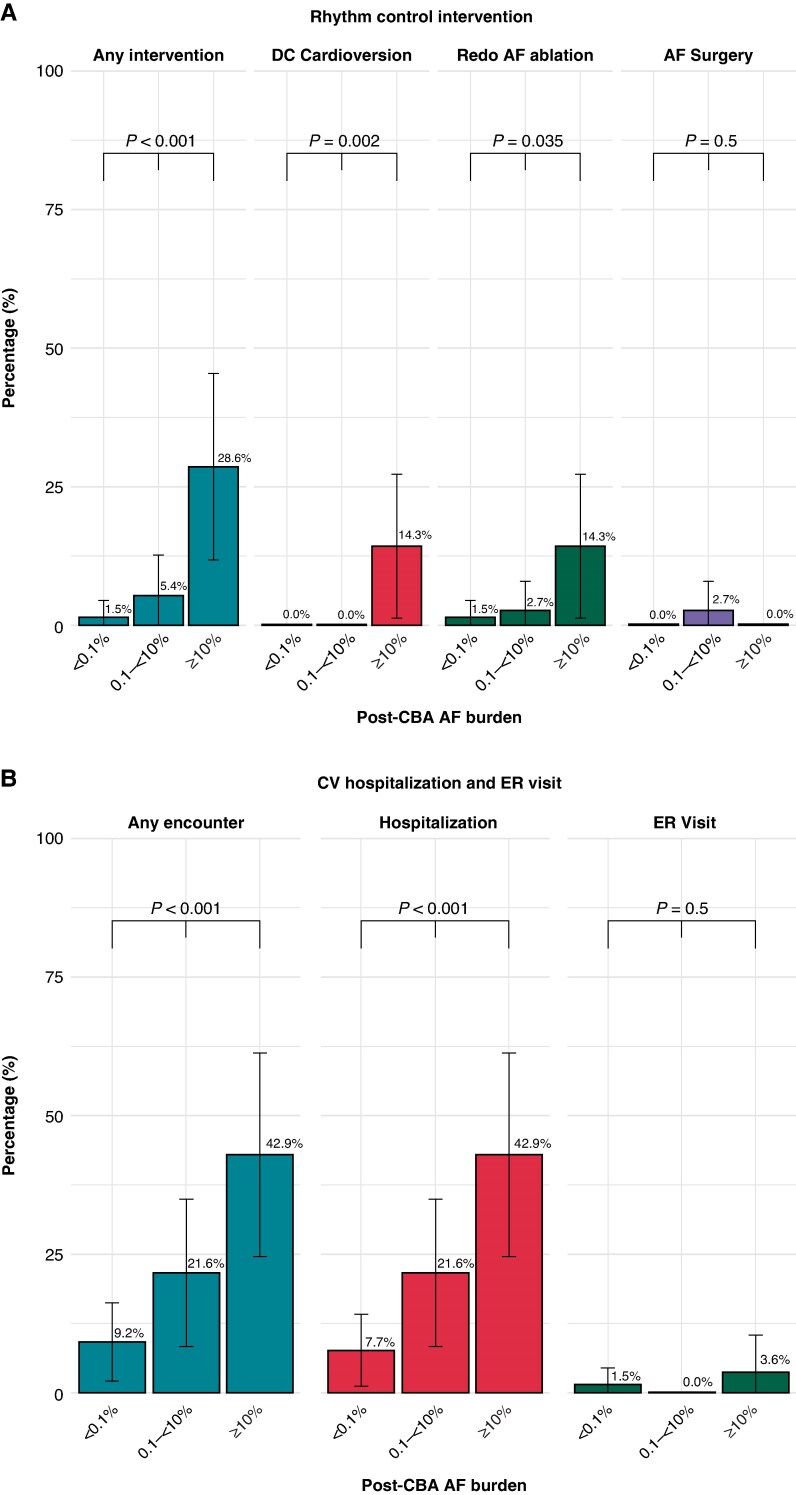
Healthcare resource utilization during 1-year follow-up post-CBA. Proportion of patients requiring atrial fibrillation–related healthcare resource utilization (HCRU) stratified by post-cryoablation (CBA) atrial fibrillation (AF) burden. Rhythm control interventions were counted only if performed after the 90-day post-ablation blanking period. The ≥10% group had significantly higher utilization of rhythm control interventions (29%) compared to the 0.1% to <10% (5.4%) and <0.1% (1.5%) groups (*P* < 0.001). This included a higher rate of direct current cardioversion (DCC, 14%) and redo AF ablation (14%) in the ≥10% group (*P* < 0.001 and *P* = 0.035, respectively). For cardiovascular (CV)-related hospitalizations and emergency room (ER) visits, the ≥10% group had a higher rate (43%) than the 0.1 to <10% group (22%) and the <0.1% group (9.2%) (*P* < 0.001). Hospitalizations were 43%, 22%, and 7.7%, and ER visits were 3.6%, 0%, and 1.5% in the ≥10%, 0.1 to <10%, and <0.1% groups, respectively (*P* < 0.001 and *P* = 0.5).

*Figure 3* shows the proportion of patients who required AF-related HCRU. Between the 90-day post-ablation blanking period and the 1-year follow-up, all types of rhythm control interventions were more common in the ≥10% post-CBA burden group (29%) than the 0.1 to <10% (5.4%) and <0.1% (1.5%) groups (*P* < 0.001). Specifically, the rates of DCC and redo AF ablation in the ≥10% group were both 14%, compared to 0% and 2.7% in the 0.1 to <10% group and 0% and 1.5% in the <0.1% group, respectively (*P* < 0.001 for DCC; *P* = 0.035 for repeat ablation).

CV-related hospitalizations occurred in 43% of patients in the ≥10% group, 22% in the 0.1 to <10% group, and 7.7% in the <0.1% group (*P* < 0.001). CV-related ER visits were reported in 3.6% of patients in the ≥10% group and 1.5% in the <0.1% group; no cases were observed in the 0.1 to <10% group (*P* = 0.5). For any encounters, the ≥10% group (43%) had higher rates than the 0.1 to <10% group (22%) and the <0.1% group (9.2%) (*P* < 0.001).

When the 90-day post-ablation blanking period was included (see Supplementary material online, *Figure S3*), the proportions of patients undergoing DCC increased to 48% in the ≥10% group, 11% in the 0.1 to <10% group, and 3.3% in the <0.1% group. The proportions of Redo AF ablation and AF surgery both increased to 5.4% in the 0.1 to <10% group.

### Symptom, quality-of-life changes, and atrial fibrillation–related healthcare resource utilization according to post-cryoballoon ablation atrial fibrillation burden by pre-cryoballoon ablation subtypes of persistent atrial fibrillation

At 1-year post-CBA, the mean AF burden significantly decreased in both the high-burden paroxysmal AF group (from 24.5 ± 19.2% to 3.7 ± 17.4%, *P* < 0.001) and the ILR-confirmed persistent AF group (from 95.4 ± 11.9% to 16.2 ± 31.1%, *P* < 0.001) (see Supplementary material online, *Table S3***)**. Among 33 patients with high-burden paroxysmal AF, 22 (66%) had a post-CBA AF burden of <0.1%, 9 (27%) had 0.1 to <10%, and 2 (6%) had ≥10%. The corresponding changes in mean AF burden were 20.8 ± 15.7% to 0.0 ± 0.0% (*P* < 0.001), 32.5 ± 23.2% to 1.2 ± 1.3% (*P* = 0.004), and 28.3 ± 37.5% to 55.4 ± 63.1% (*P* = 0.5), respectively, with a statistically significant difference among the groups (*P* = 0.034). Among 97 patients with ILR-confirmed persistent AF, 43 (44%) had a post-CBA AF burden of <0.1%, 28 (29%) had 0.1 to <10%, and 26 (27%) had ≥10%. Their mean AF burden changed from 97.8 ± 7.1% to 0.0 ± 0.0% (*P* < 0.001), 91.3 ± 17.5% to 1.4 ± 2.3% (*P* < 0.001), and 95.7 ± 10.0% to 58.9 ± 33.5% (*P* < 0.001), respectively, with significant differences between the groups (*P* < 0.001).

The proportion of patients reporting symptom improvement was numerically higher in the high-burden paroxysmal AF group than in the ILR-confirmed persistent AF group (85% vs. 74%, *P* = 0.13) (see Supplementary material online, *Table S3* and Supplementary material online, *Figure S4*). Among patients with high-burden paroxysmal AF, symptom improvement was reported in 95%, 67%, and 50% of those with post-CBA AF burdens of <0.1%, 0.1 to <10%, and ≥10%, respectively (*P* = 0.048). Similarly, in the ILR-confirmed persistent AF group, symptom improvement was observed in 86%, 82%, and 46% of patients with post-CBA AF burdens of <0.1%, 0.1 to <10%, and ≥10%, respectively (*P* < 0.001).

QoL improvements followed trends observed in the overall study population. Both the high-burden paroxysmal AF group and the ILR-confirmed persistent AF groups exhibited significant increases in total SF-36 (24.1 ± 32.8 and 14.4 ± 30.1), PCS (14.2 ± 17.4 and 7.6 ± 16.8), and MCS scores (9.9 ± 19.2 and 6.9 ± 16.1) (all *P* < 0.01) (see Supplementary material online, *Table S3*). The differences between the two groups were not significant (*P* = 0.2, 0.2, and 0.3 for total SF-36, PCS, and MCS, respectively). Within the high-burden paroxysmal AF group, only the <0.1% burden subgroup demonstrated significant QoL improvement, and in the ILR-confirmed persistent AF group, the <0.1% and 0.1% to <10% subgroups experienced significant QoL improvements.

Regarding AF-related HCRU, all rhythm control interventions occurred in the ILR-confirmed persistent AF group, while only one patient in the high-burden paroxysmal AF group required CV-related hospitalization and an ER visit.

## Discussion

This study comprehensively examined the quantitative association between continuously monitored 1-year post-CBA AF burden and the improvement of AF-related symptoms, QoL, and HCRU. We found that in early persistent AF patients with a mean pre-CBA AF burden of 77%, CBA significantly reduced AF burden, with 50.0% achieving a 1-year post-CBA AF burden of <0.1%, 28.5% falling within the 0.1 to <10% range, and 21.5% remaining at ≥10%. Stratification revealed that lower-burden groups experienced greater AF-related symptom improvement, significant QoL enhancement, and reduced HCRU. Furthermore, while achieving a zero per cent AF burden post-CBA offers the best outcomes, substantial burden reduction to below 10% still provides meaningful clinical benefits in AF-related symptom relief, QoL improvement, and decreased HCRU.

### Atrial fibrillation burden assessment and monitoring

Several prior studies have demonstrated the positive effects of PVI on AF-related symptoms, QoL, and HCRU.^3,22–25^ Furthermore, recent studies have suggested that AF burden assessment may offer advantages over the conventional binary endpoint of AF recurrence in predicting post-ablation clinical outcomes.^12,13^ Trials have shown that reductions in AF burden are associated with improvements in symptoms and QoL after PVI. However, the ability to quantitatively evaluate this relationship is highly dependent on the monitoring modality used. Most earlier studies relied on intermittent monitoring tools such as Holter monitors, transtelephonic electrocardiogram monitors (TTMs), or daily electrocardiogram strips, which often fail to capture a substantial proportion of AF episodes and lead to unreliable burden estimates.^26–29^

For instance, the STAR AF II trial found that persistent AF patients with <70% burden reduction after PVI exhibited attenuated improvement in PCS scores.^30^ The ADVENT trial reported greater QoL improvements and lower HCRU in patients with post-ablation AF burden ≥10% than those with <0.1%.^12^ Similarly, DECAAF II and PULSED-AF trials showed a strong correlation between post-ablation burden reduction and symptom or QoL improvement.^14,31^ However, these findings are constrained by their reliance on intermittent monitoring, which limits the precision of AF burden quantification.

Implantable cardiac monitors, in contrast, offer greater sensitivity for AF detection and provide more accurate and continuous AF burden data.^32,33^ Even in patients with high AF burden, intermittent monitoring can yield burden measurement errors exceeding 20%.^16^ In our study, 25% of patients classified as having clinically persistent AF were found to have high-burden paroxysmal AF when monitored continuously. Recognizing the limitations of intermittent monitoring, a prior study proposed a proxy method using noninvasive, smartphone app-based rhythm assessments to approximate continuously monitored AF burden.^34^ Our study exclusively used ILR to estimate both pre- and post-CBA burden and detect recurrent AF, providing the most precise evaluation of post-ablation burden. This approach allowed us to demonstrate a robust quantitative relationship between AF burden reduction and all three key outcomes: AF-related symptoms, QoL, and HCRU.

We chose to assess the post-CBA AF burden at 1 year to more accurately reflect the final AF burden at the time of patient visits and surveys, rather than using the burden over the entire post-CBA period. Patients in the <0.1% burden group had no burden at all. Among patients with recurrent AF at 1 year, 60% of those in the 0.1 to <10% group had minimal AF burden (mean: 1.34%), while 40% in the ≥10% group had a moderately high AF burden (mean: 59%). The ≥10% group had a longer duration in persistent AF and a higher proportion of ILR-confirmed persistent AF, suggesting that the cumulative effects of AF progression and structural remodelling may influence both CBA effectiveness and post-CBA AF burden.

### Atrial fibrillation–related symptom improvement

In the overall population, AF burden significantly decreased in all three burden groups, accompanied by corresponding improvements in EHRA symptom scores. Baseline symptom severity was similar among the three groups, with most patients experiencing class IIa or higher symptoms. However, post-CBA symptom improvement varied depending on AF burden. In the <0.1% group, the majority of patients became symptom-free, whereas approximately 60% achieved symptom resolution in the 0.1 to <10% group. In contrast, only 25% of patients in the ≥10% group were symptom-free, and about 10% reported worsened symptoms. These findings strongly support the well-established relationship between AF burden and AF-related symptoms.^35,36^ Prior studies have demonstrated that reducing AF burden, whether through catheter ablation or medical rhythm control, correlates with symptom improvement, including in persistent AF.^3,31,37,38^ And our study reinforces these findings in the context of CBA, particularly in early persistent AF. Additionally, HCRU data indicate that DCC and redo ablation were predominantly performed in high-burden patients, further supporting the symptomatic burden relationship with post-CBA AF burden.

### Quality-of-life outcomes

Significant QoL improvement at 1-year post-CBA was observed only in patients with lower post-CBA burden (<0.1% and 0.1 to <10%). The improvement in both PCS and MCS scores was numerically greater in these patients than in those with higher post-CBA burden, though direct comparisons were not statistically significant. These findings align with the pattern of AF burden reduction: while all three groups experienced burden reduction, the magnitude of reduction was greater in the lower-burden groups. They also align with previous studies that have consistently demonstrated a strong correlation between AF symptom severity and QoL.^39^ However, as a generic QoL instrument, the SF-36 may be less sensitive to changes specific to AF symptoms and burden compared to AF-specific QoL scales. This limited sensitivity may partly explain the lack of statistically significant differences across burden groups.

Our study also revealed a similar relationship between symptom improvement and QoL improvement, alongside corresponding burden reduction (see Supplementary material online, *Figure S5*). However, the high variability in QoL scores and the less clear association with burden reduction indicate the complex and multifactorial nature of QoL. In patients with AF, QoL is influenced not only by AF-related symptoms but also by complications from interventions, patient expectations for favourable outcomes, fear of future adverse events, and financial concerns.^40^ These diverse patient factors should be carefully considered when interpreting QoL changes following ablative therapy in AF patients.

### Healthcare resource utilization

HCRU findings suggest that post-CBA AF burden in early persistent AF serves as a practical indicator of downstream clinical resource use. Higher burden was associated with more frequent DCC, redo ablation, and CV-related hospitalizations, highlighting the clinical and potential economic impact of residual AF burden.

In addition, OAC use at 12 months was also higher among patients with higher post-CBA AF burden. In multivariable regression analysis, post-CBA AF burden was positively associated with continued OAC use (see Supplementary material online, *Tables S4* and *S5*), particularly among patients with low thromboembolic risk (CHA_2_DS_2_-VASc score ≤1 in men and ≤2 in women). These findings suggest that continuously monitored AF burden may influence physicians’ decisions regarding OAC discontinuation.

### Implications for clinical practice and future research

A key strength of this study is its comprehensive assessment of pre- and post-CBA AF burden in early persistent AF patients, making it the first study to correlate continuously monitored post-CBA AF burden with AF-related symptoms, QoL, and HCRU. A few recent trials have employed implantable cardiac monitors to explore the association. The CIRCA-DOSE trial demonstrated burden-related improvements in QoL and reductions in HCRU among patients with paroxysmal AF undergoing thermal ablation.^13^ In persistent AF, the ADVANTAGE-AF Phase II trial reported increased HCRU in patients with post-ablation burden >0.1% following pulsed-field ablation.^41^ However, it did not identify a clear burden threshold associated with superior QoL improvement, possibly due to the larger absolute burden reduction in persistent AF compared to paroxysmal AF. Our findings align with these studies and further suggest that, even in persistent AF, the extent of residual AF burden may influence the degree of QoL improvement.

Our study stratified continuous AF burden into <0.1% (0%), 0.1 to <10%, and ≥10%, revealing that even patients with post-PVI burden below 10%—not only those achieving zero per cent—experienced significant improvements in symptoms, QoL, and reductions in HCRU. These findings suggest that in persistent AF, treatment evaluation should extend beyond the binary concept of AF recurrence and adopt a continuous burden perspective, where a clinically acceptable recurrence burden may exist. This evolving view reflects the broader evolution of catheter ablation techniques over the past 25 years.^42^ The development of more durable PVI tools—regardless of energy source—and an improved understanding of arrhythmia mechanisms have shifted the focus of AF ablation beyond acute procedural success and binary recurrence status, towards more sustained rhythm control and patient-centred outcomes. Our findings align with this trend, showing that in early persistent AF, a reduction in AF burden may be associated with improvements in symptoms, quality of life, and healthcare utilization. Although our study used CBA, the concept of burden-based assessment may be applicable across different PVI strategies, including radiofrequency and pulsed-field ablation. These results suggest that AF burden could complement recurrence-based endpoints when evaluating the effectiveness of ablation in persistent AF. Further studies are warranted to define clinically relevant burden thresholds and to support the broader adoption of burden-based outcomes in clinical practice.

### Study limitations

This study has several limitations. First, we used EHRA symptom scores and SF-36 surveys as the sole assessment tools for symptoms and QoL, which may limit the generalizability of our findings and the comparability with studies that used different or multiple assessment tools. In addition, the SF-36 may be less sensitive to changes specific to AF symptoms and burden, potentially underestimating the impact of AF burden reduction. Second, the lack of a control group in this single-arm study prevents validation of symptom and QoL improvements against a comparison group. Third, the timing of ILR implantation varied between 7 and 90 days before CBA, which may have influenced the accurate calculation of pre-CBA AF burden and the classification of AF subtype. Finally, the study population was exclusively Korean, which may limit the generalizability of these findings to other ethnic groups and healthcare settings.

## Conclusions

Patients with early persistent AF showed that groups with lower AF burden experienced greater improvements in AF-related symptoms, statistically significant enhancements in QoL, and reduced HCRU during follow-up. Notably, while a zero per cent AF burden post-PVI is optimal, reducing AF burden below 10% still provides meaningful clinical benefits. These findings underscore the importance of post-PVI AF burden reduction in improving patient outcomes and minimizing the need for further interventions.

## Supplementary Material

euaf150_Supplementary_Data

## Data Availability

The datasets generated and analysed during this study are available from the corresponding author upon reasonable request.
