# Supplementary material for: Atrial fibrillation burden and symptom, quality of life, and healthcare resource utilization after cryoballoon ablation in persistent atrial fibrillation
Source: Europace. 2025 Aug 29;27(8):euaf150. doi: 10.1093/europace/euaf150 (PMC12395340; doi:10.1093/europace/euaf150)

Supplementary Table S1. Procedural details of cryoballoon ablation and implantable loop recorder programming.

Cryoballoon ablation procedure	Implantable loop recorder programming
· Anesthesia & Access: Conscious sedation or general anesthesia was used. Transseptal puncture was performed via femoral venous access to reach the left atrium (LA).<br>· Heparin Administration: Given post-transseptal puncture to keep activated clotting time above 300 seconds.<br>· Cryoballoon Ablation: A 28-mm cryoballoon (Medtronic Arctic Front Advance) and mapping catheter were used to isolate pulmonary veins (PVI). Cryoapplication duration was typically 240 seconds per vein with a freeze-thaw-freeze technique.<br>· Phrenic Nerve Monitoring: Diaphragmatic movement was monitored during right pulmonary vein ablation to avoid nerve injury.<br>· Endpoint: The goal was a bidirectional conduction block in all veins, confirmed after a 20-minute observation period; repeat ablation if reconnection was detected.<br>· Adjunct Procedures: Electrical/pharmacologic cardioversion for sinus rhythm if needed. Radiofrequency ablation for right atrial flutter if necessary.<br>· Post-procedure Management: Systemic anticoagulation for at least 2 months. Discontinuation or adjustment of antiarrhythmic drugs (AADs) was at physician discretion after a 90-day blanking period.	· AF detection threshold: balanced sensitivity<br>· Ectopy rejection: nominal<br>· Episode storage threshold: all<br>*These parameters were chosen to optimize detection of AF (reported sensitivity of 96.1% with a positive predictive value of 73%) [1,2]. All arrhythmia episodes recorded by the implantable loop recorder were adjudicated by investigators (EKC, SRL, and JMC).

***References**

[1] Mittal S, Rogers J, Sarkar S, et al. Real-world performance of an enhanced atrial fibrillation detection algorithm in an insertable cardiac monitor. Heart Rhythm 2016;13:1624-30.

[2] Sanders P, Purerfellner H, Pokushalov E, et al. Performance of a new atrial fibrillation detection algorithm in a miniaturized insertable cardiac monitor: Results from the Reveal LINQ Usability Study. Heart Rhythm 2016;13:1425-30.

Supplementary Table S2. Inclusion and exclusion criteria of the study.

Inclusion criteria	Exclusion criteria
1) Patients aged 20 to 80 years<br>2) drug-refractory symptomatic persistent AF ≤3 years<br>3) Persistent AF Definition:<br>· Documented AF on 12-lead ECGs taken at least 7 days apart.<br>· 100% AF burden confirmed by a 24-hour Holter monitor.<br>· Requires cardioversion for AF episodes lasting ≥7 days.	1) Long-standing persistent AF more than 3-year<br>2) Sinus rhythm at enrollment<br>3) Recurrent sinus rhythm after electrical cardioversion<br>4) Severe left ventricular dysfunction (left ventricular ejection fraction <30%)<br>5) Previous ablation procedure or surgery for atrial fibrillation<br>6) Contraindication to chronic anticoagulation therapy or heparin<br>7) Documented left atrial diameter >50mm from parasternal long-axis view<br>8) A percutaneous coronary intervention or myocardial infarction ≤ 3 months<br>9) A stroke or transient ischemic attack <6 months<br>10) Planned cardiovascular intervention<br>11) Mental or physical inability to participate in the study<br>12) Participation in another randomized clinical trial<br>13) Uncontrolled hypertension, untreated hypothyroidism, or hyperthyroidism<br>14) Requirement for dialysis due to terminal renal failure

Supplementary Table S3. Post-ablation AF burden and SF-36, EHRA symptom score change compared by persistent AF subtypes according to 1-year post-CBA AF burden.

AF subtype	Post-CBA AF burden	n (%)	Pre-CBA burden	Post-CBA burden	Burden change	EHRA symptom improvement (proportion)	QoL improvement (Total SF-36)	QoL improvement (PCS)	QoL improvement (MCS)
High-burden paroxysmal AF	Total	33 (100%)	24.5 (19.2)<br>23.9 [6.4, 35.9]	3.7 (17.4)<br>0.0 [0.0, 0.2]	-20.8 (22.0)<br>-21.1 [-33.7, -5.9]	28 (85%)	24.1 (32.8)	14.2 (17.4)	9.9 (19.2)
	<0.1%	22 (66%)	20.8 (15.7)<br>20.8 [6.5, 29.1]	0.0 (0.0)<br>0.0 [0.0, 0.0]	-20.8 (15.7)<br>-20.8 [-29.1, -6.5]	21 (95%)	26.8 (30.7)	14.3 (16.3)	12.5 (17.8)
	0.1 to <10%	9 (27%)	32.5 (23.2)<br>35.6 [9.3, 46.8]	1.2 (1.3)<br>0.5 [0.2, 2.3]	-31.2 (22.8)<br>-32.3 [-46.4, -8.8]	6 (67%)	15.1 (38.3)	15.0 (21.7)	0.1 (19.0)
	≥10%	2 (6%)	28.3 (37.5)<br>28.3 [15.0, 41.5]	55.4 (63.1)<br>55.4 [33.1, 77.7]	27.2 (25.5)<br>27.2 [18.1, 36.2]	1 (50%)	35.6 (41.6)	9.4 (15.9)	26.2 (25.7)
	p-value		0.5	<0.001	0.034	0.048	0.7	0.9	0.4
ILR-confirmed persistent AF	Total	97 (100%)	95.4 (11.9)<br>100.0 [98.4, 100.0]	16.2 (31.1)<br>0.1 [0.0, 14.3]	-79.2 (33.1)<br>-99.2 [-100.0, -76.2]	72 (74%)	14.4 (30.1)	7.6 (16.8)	6.9 (16.1)
	<0.1%	43 (44%)	97.8 (7.1)<br>100.0 [99.9, 100.0]	0.0 (0.0)<br>0.0 [0.0, 0.0]	-97.8 (7.1)<br>-100.0 [-100.0, -99.9]	37 (86%)	13.9 (24.5)	7.6 (15.4)	6.3 (12.2)
	0.1 to <10%	28 (29%)	91.3 (17.5)<br>100.0 [94.5, 100.0]	1.4 (2.3)<br>0.4 [0.1, 1.0]	-89.9 (17.4)<br>-99.0 [-99.7, -90.8]	23 (82%)	20.0 (34.9)	9.9 (18.2)	10.1 (19.9)
	≥10%	26 (27%)	95.7 (10.0)<br>99.9 [96.7, 100.0]	58.9 (33.5)<br>64.8 [21.0, 93.7]	-36.9 (34.7)<br>-28.0 [-76.1, -1.0]	12 (46%)	9.4 (33.0)	5.0 (17.9)	4.3 (17.4)
	p-value		0.2	<0.001	<0.001	<0.001	0.4	0.5	0.4

AF: atrial fibrillation; CBA: cryoballoon ablation; EHRA: European Heart Rhythm Association; ILR: implanatable loop recorder; MCS: mental component score; PCS: physical component score; QoL: quality of life

Supplementary Table S4. Post-CBA AF burden and oral anticoagulant use at 12-month follow-up.

	Total (n=126)			Male (n=96)			Female (n=30)		
Post-CBA AF burden	n	No OAC	OAC	n	No OAC	OAC	n	No OAC	OAC
CHA_2DS_2-VASc score									
<1 (Male), <2 (Female)	33	23 (70%)	10 (30%)	26	20 (77%)	6 (23%)	7	3 (43%)	4 (57%)
<0.1%	20	18 (90%)	2 (10%)	17	16 (94%)	1 (5.9%)	3	2 (67%)	1 (33%)
0.1% to <10%	6	4 (67%)	2 (33%)	5	3 (60%)	2 (40%)	1	1 (100%)	0 (0.0%)
≥10%	7	1 (14%)	6 (86%)	4	1 (25%)	3 (75%)	3	0 (0.0%)	3 (100%)
p-value	<0.001			0.009			0.229		
1 (Male), 2 (Female)	32	10 (31%)	22 (69%)	22	9 (41%)	13 (59%)	10	1 (10%)	9 (90%)
<0.1%	11	5 (45%)	6 (55%)	8	4 (50%)	4 (50%)	3	1 (33%)	2 (67%)
0.1% to <10%	11	5 (45%)	6 (55%)	7	5 (71%)	2 (29%)	4	0 (0.0%)	4 (100%)
≥10%	10	0 (0.0%)	10 (100%)	7	0 (0.0%)	7 (100%)	3	0 (0.0%)	3 (100%)
p-value	0.035			0.028			0.6		
≥2 (Male), ≥3 (Female)	61	3 (4.9%)	58 (95%)	48	3 (6.3%)	45 (94%)	13	0 (0.0%)	13 (100%)
<0.1%	32	2 (6.3%)	30 (94%)	27	2 (7.4%)	25 (93%)	5	0 (0.0%)	5 (100%)
0.1% to <10%	19	1 (5.3%)	18 (95%)	14	1 (7.1%)	13 (93%)	5	0 (0.0%)	5 (100%)
≥10%	10	0 (0.0%)	10 (100%)	7	0 (0.0%)	7 (100%)	3	0 (0.0%)	3 (100%)
p-value	1.0			1.0			-		

AF: atrial fibrillation; CBA: cryoballoon ablation; OAC: oral anticoagulant.
p-values were calculated using Fisher's exact test.

Supplementary Table S5. Coefficients from multivariable regression analysis of oral anticoagulant use at 12-month follow-up.

	Coefficient	Standard Error	z value	P-value	Odds ratio (95% CI)
(Intercept)	-10.1310	4.7496	-2.1330	0.033	0.000 (0.000 – 0.210)
Post-CBA AF Burden					
0.1 to <10%	0.2969	0.7243	0.4100	0.682	1.346 (0.320 – 5.753)
≥10%	5.4064	1.5739	3.4350	0.001	222.8 (15.92 – 9369.8)
Age	0.1279	0.0503	2.5420	0.011	1.136 (1.039 – 1.269)
Sex (Female)	-0.7074	0.9606	-0.7360	0.461	0.493 (0.072 – 3.360)
Body-mass index (per kg/m^2)	0.0313	0.1337	0.2340	0.815	1.032 (0.797 – 1.361)
CHA_2DS_2-VASc score	2.2026	0.4780	4.6080	0.000	9.049 (3.965 – 26.68)
Rhythm control interventions	-1.1215	0.6157	-1.8210	0.069	0.326 (0.086 – 1.194)
Antiarrhythmic use	-0.7296	0.7311	-0.9980	0.318	0.482 (0.103 – 1.910)

AF: atrial fibrillation; CBA: cryoballoon ablation; CI: confidence interval.

	Coefficient	Standard Error	z value	P-value	Odds ratio (95% CI)
(Intercept)	-10.3557	4.9873	-2.0760	0.038	0.000 (0.000 – 0.236)
Post-CBA AF Burden (continuous)	0.2810	0.0953	2.9500	0.003	1.325 (1.124 – 1.647)
Age	0.1338	0.0513	2.6100	0.009	1.143 (1.044 – 1.278)
Sex (Female)	-0.6753	0.9947	-0.6790	0.497	0.509 (0.068 – 3.690)
Body-mass index (per kg/m^2)	0.0197	0.1391	0.1410	0.888	1.020 (0.783 – 1.363)
CHA_2DS_2-VASc score	2.3209	0.5126	4.5280	0.000	10.19 (4.234 – 32.75)
Rhythm control interventions	-1.4319	0.6857	-2.0880	0.037	0.239 (0.051 – 0.894)
Antiarrhythmic use	-0.6548	0.7397	-0.8850	0.376	0.520 (0.111 – 2.125)

AF: atrial fibrillation; CBA: cryoballoon ablation; CI: confidence interval.

Supplementary Figure S1. Study enrollment flow.

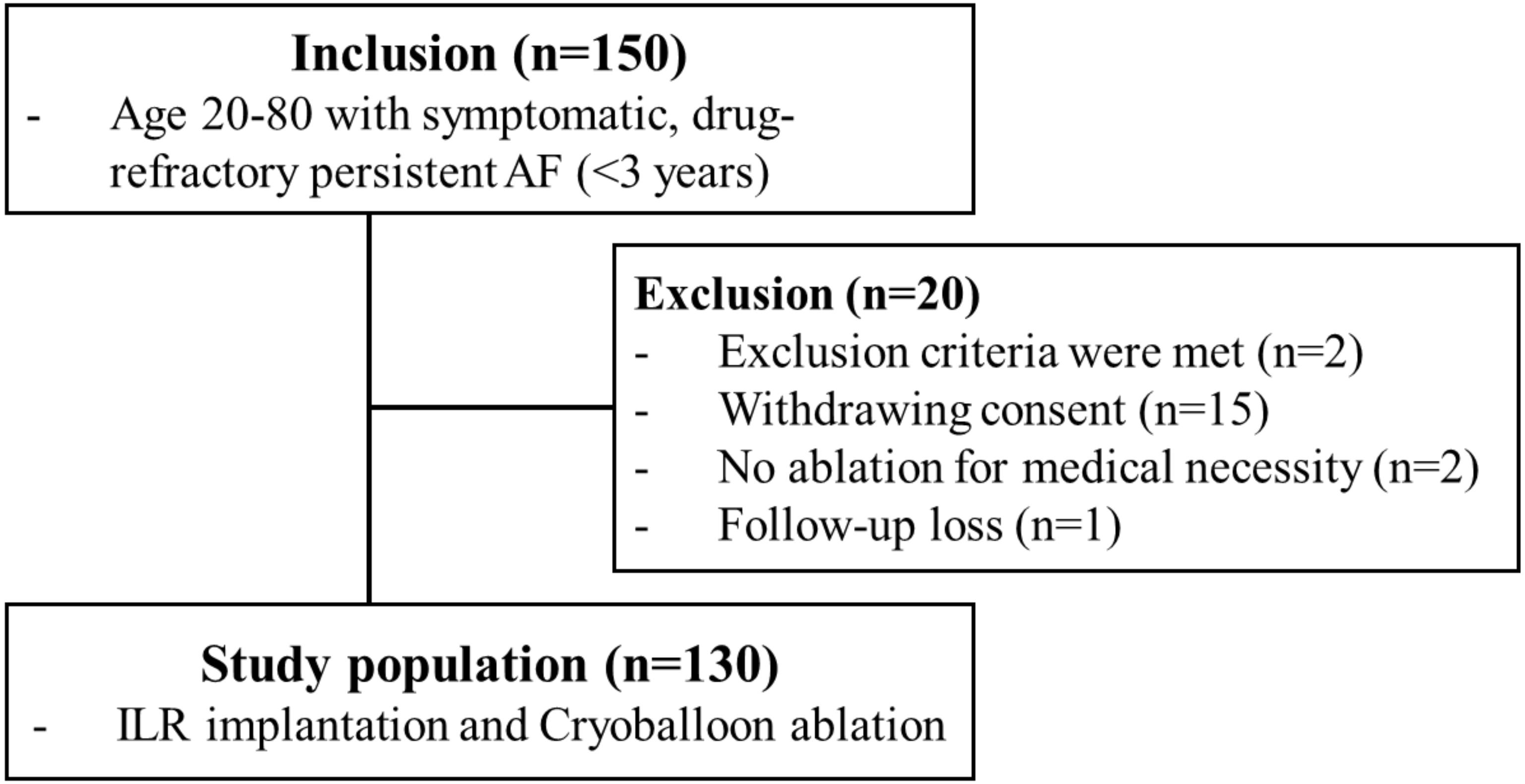

Supplementary Figure S2. Pre-/Post-ablation ILR AF burden in study population.

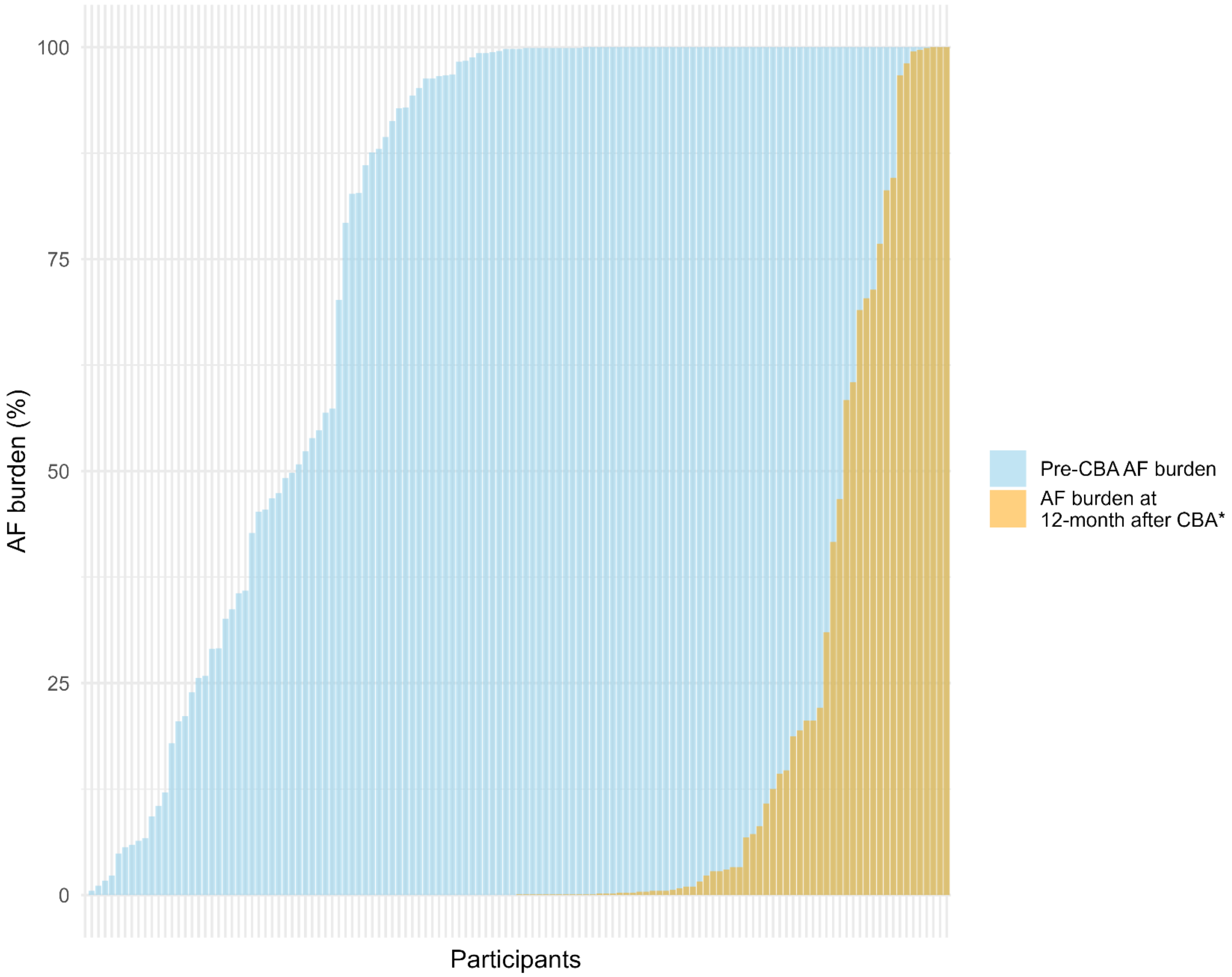

*AF burden monitored between 9-month to 12-month after CBA

Supplementary Figure S3. Rhythm control interventions during 1-year follow-up post-CBA (including blanking period).

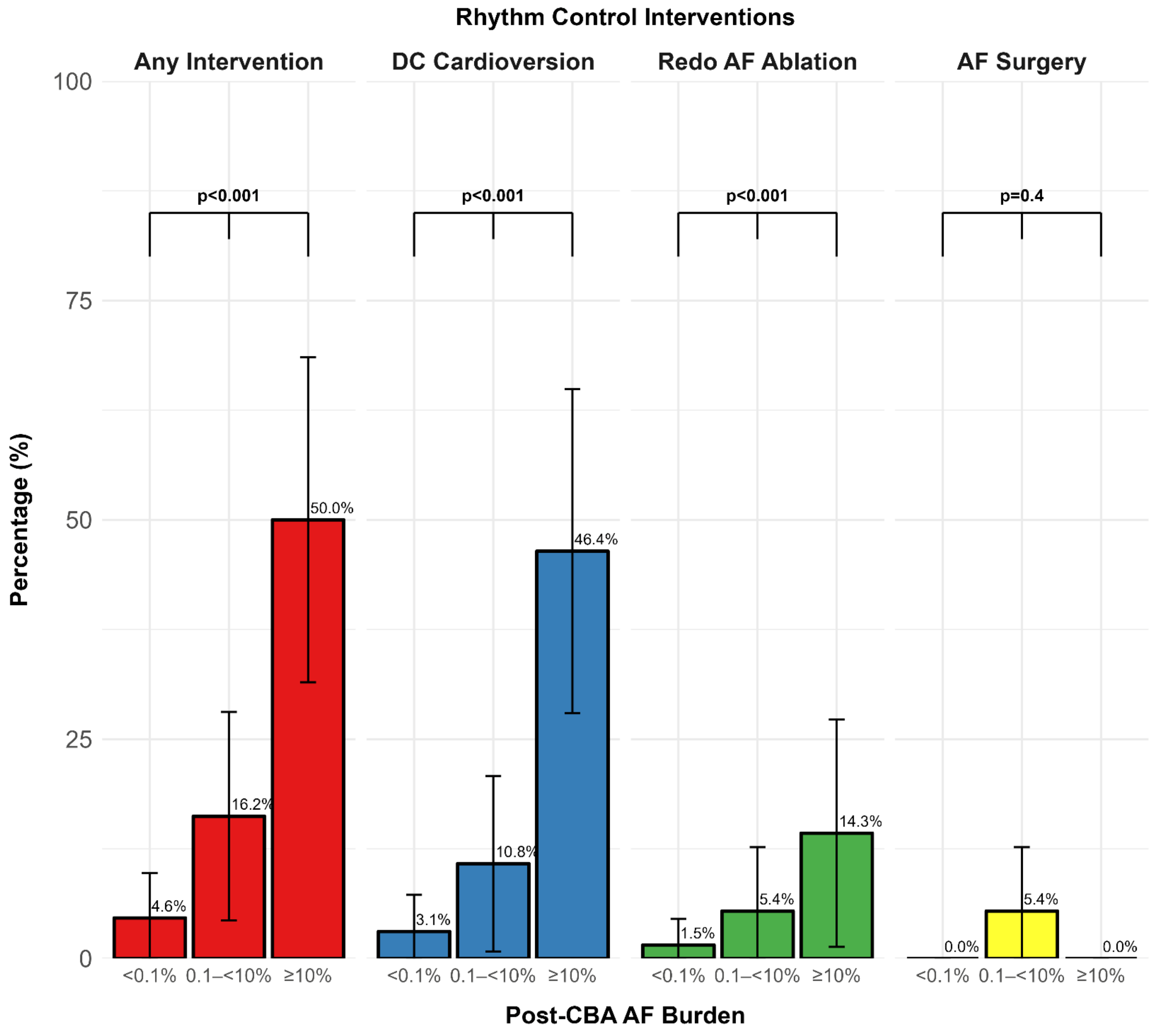

Supplementary Figure S4. Symptom changes before and after CBA by 1-year post-CBA AF burden (based on pre-CBA AF subtypes).

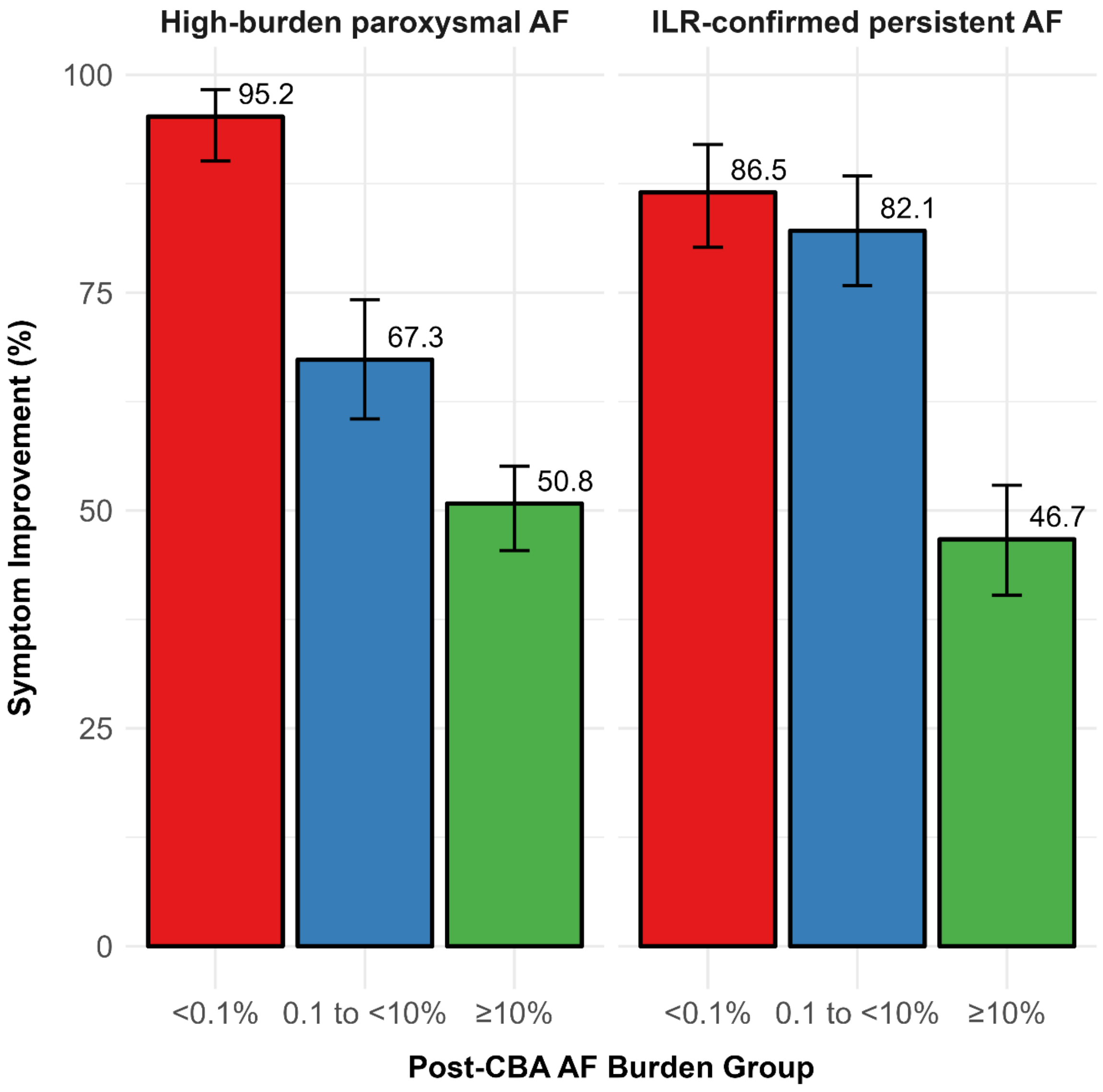

Supplementary Figure S5. Association between symptom improvement and quality of life gains.

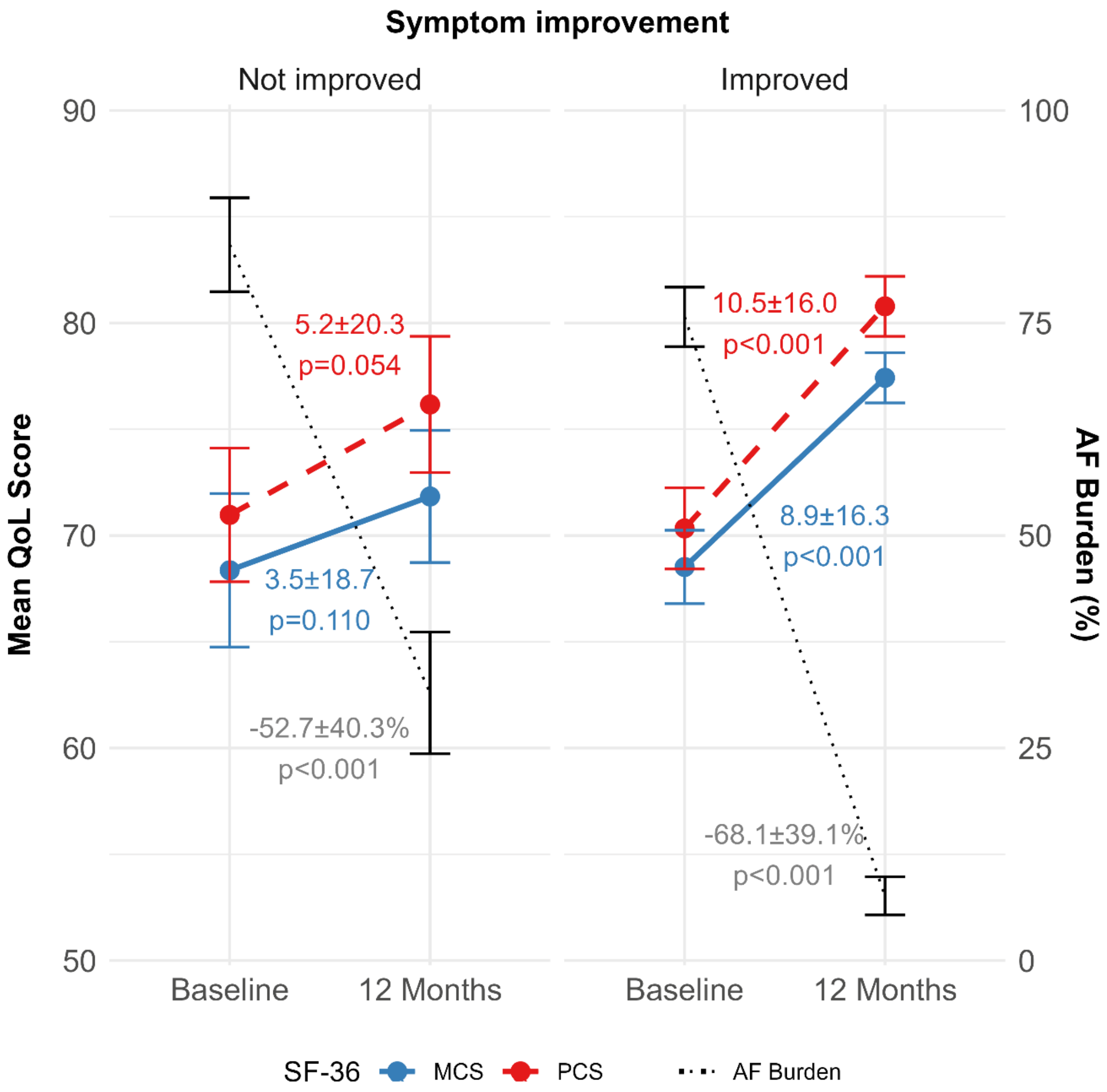

Supplement: euaf150_Supplementary_Data [file euaf150_supplementary_data.pdf]
